# Environmental Drivers of *Culicoides* Phenology: How Important Is Species-Specific Variation When Determining Disease Policy?

**DOI:** 10.1371/journal.pone.0111876

**Published:** 2014-11-11

**Authors:** Kate R. Searle, James Barber, Francesca Stubbins, Karien Labuschagne, Simon Carpenter, Adam Butler, Eric Denison, Christopher Sanders, Philip S. Mellor, Anthony Wilson, Noel Nelson, Simon Gubbins, Bethan V. Purse

**Affiliations:** 1 Centre for Ecology and Hydrology, Bush Estate, Penicuik, Midlothian, United Kingdom; 2 The Pirbright Institute, Ash Road, Pirbright, Woking, United Kingdom; 3 PVVD, ARC-Onderstepoort Veterinary Institute, Private Bag X05, Onderstepoort, South Africa; 4 Biomathematics and Statistics Scotland, James Clerk Maxwell Building, The King’s Buildings, Edinburgh, United Kingdom; 5 The Met Office, Based at The Pirbright Institute, Pirbright, Woking, Surrey, United Kingdom; University of Thessaly, Greece

## Abstract

Since 2006, arboviruses transmitted by *Culicoides* biting midges (Diptera: Ceratopogonidae) have caused significant disruption to ruminant production in northern Europe. The most serious incursions involved strains of bluetongue virus (BTV), which cause bluetongue (BT) disease. To control spread of BTV, movement of susceptible livestock is restricted with economic and animal welfare impacts. The timing of BTV transmission in temperate regions is partly determined by the seasonal presence of adult *Culicoides* females. Legislative measures therefore allow for the relaxation of ruminant movement restrictions during winter, when nightly light-suction trap catches of *Culicoides* fall below a threshold (the ‘seasonally vector free period’: SVFP). We analysed five years of time-series surveillance data from light-suction trapping in the UK to investigate whether significant inter-specific and yearly variation in adult phenology exists, and whether the SVFP is predictable from environmental factors. Because female vector *Culicoides* are not easily morphologically separated, inter-specific comparisons in phenology were drawn from male populations. We demonstrate significant inter-specific differences in *Culicoides* adult phenology with the season of *Culicoides scoticus* approximately eight weeks shorter than *Culicoides obsoletus.* Species-specific differences in the length of the SVFP were related to host density and local variation in landscape habitat. When the *Avaritia Culicoides* females were modelled as a group (as utilised in the SFVP), we were unable to detect links between environmental drivers and phenological metrics. We conclude that the current treatment of *Avaritia Culicoides* as a single group inhibits understanding of environmentally-driven spatial variation in species phenology and hinders the development of models for predicting the SVFP from environmental factors. *Culicoides* surveillance methods should be adapted to focus on concentrated assessments of species-specific abundance during the start and end of seasonal activity in temperate regions to facilitate refinement of ruminant movement restrictions thereby reducing the impact of *Culicoides*-borne arboviruses.

## Introduction

Northern Europe is currently experiencing an unprecedented series of incursions of arboviruses transmitted between ruminants by *Culicoides* (Diptera: Ceratopogonidae) [1]. Five separate strains of bluetongue virus (BTV) have been recorded in the region since 2006 [2]–[4], and a novel *Culicoides*-borne arbovirus, provisionally named Schmallenberg virus (SBV), was discovered by metagenomic studies in Germany in 2011 [5], following unexplained clinical signs in dairy cattle. SBV has since spread rapidly across a large geographic area, and has been found to inflict foetal abnormalities in both cattle and sheep [6]. The route of entry of several of these arbovirus strains remains undefined [7]; hence the risk of further emergence of *Culicoides*-borne pathogens in this region cannot easily be estimated or mitigated.

In temperate ecosystems, the seasonal incidence and abundance of adult female *Culicoides* is a key parameter in determining the timing and intensity of arbovirus outbreaks and varies widely across geographical space [8]. It is thought that livestock-associated *Culicoides* in northern Europe overwinter in their final (fourth) larval instar and do not generally survive the winter as adults [9]. While live *Culicoides* adults have been recovered from animal housing in winter in northern Europe [10], [11], their numbers seem to be insufficient to drive BTV outbreaks since new confirmed clinical cases of BT are only very rarely recorded in winter (December to March).

The response to incursion of livestock arboviruses in Europe is dependent on strain pathogenicity and whether legislation exists that defines control measures. For BTV, emergence is notifiable to the World Organisation for Animal Health (OIE). Movement and trade restrictions of susceptible stock in the surrounding area are imposed immediately to limit spread of BTV (defined under Council Directive 2000/75/EC of EU legislation). Although these measures reduce the speed and extent of spread of viruses, they also impose huge logistic and welfare costs on affected regions [12] that could be minimised with enhanced understanding of geographical and annual variation in adult vector seasons.

To date, the most damaging outbreak of bluetongue (BT) in northern Europe was inflicted by a serotype 8 strain of sub-Saharan origin [1], [13]. In response to this incursion, movement restrictions were imposed across the region but vaccination to combat spread was not available until spring 2008, some eighteen months after the initial incursion was identified. In 2007, to alleviate the impact of movement restrictions, a ‘seasonal vector free period’ (SVFP) scheme was defined by the European Union council enabling movement of susceptible livestock during winter under what was hypothesised to be an extremely low risk of BTV transmission (defined in Annexe V of Commission Regulation (EC) No. 1266/2007). The declaration enabled movement of these livestock from farms in the affected zones to winter sites or markets, substantially easing the economic difficulties of farmers in the affected region and enabling some 85,000 animal movements in the UK alone during the winter of 2007 [14].

Maintenance of the SVFP is reliant upon the operation of a network of light-suction traps designed to monitor adult *Culicoides* activity [15]. In northern Europe, a threshold of catching less than five parous (abdominally pigmented) female *Culicoides* per trap was set as a limit for declaring freedom of adult activity. In some countries (e.g., the UK), this restriction was additionally underpinned using data concerning the thermal limits of BTV replication [16]. Due in part to fact that the primary vectors of BTV-8 in northern Europe were not convincingly identified to species level [1], no attempt was made to account for potential variation in phenology in *Culicoides* species that could be involved in transmission.

Unlike BTV-8, SBV has not been declared a notifiable disease by a majority of affected countries partly because substantial geographical spread had already occurred before the pathogen was first detected. For mitigating SBV impacts, it is even more imperative to understand *Culicoides* phenology, because the extent of clinical birth malformations in lambs and calves is governed by whether the ewe or dam receives an infectious *Culicoides* bite during a particular period of pregnancy [17], [18], which is close to the last portion of the adult vector season. In the event that SBV persists in this region, or in the event of emergence of more pathogenic strains, this knowledge could be employed to make alterations in husbandry practises that would reduce the impact of the disease including changes in tupping schedules [19].

In this paper light-suction trap data collected over a five year period within the UK are analysed to quantify inter-specific differences in the phenology of *Culicoides*. Phenological metrics are related to remote environmental variables with the aim of understanding how variation in the SVFP may be produced under different climate, host and landscape conditions. The livestock-associated *Culicoides* fauna in northern Europe is dominated by species belonging to the subgenus *Avaritia* and four species of this group have been identified in the UK (*Culicoides obsoletus*, *Culicoides scoticus*, *Culicoides dewulfi* and *Culicoides chiopterus*). Due to poor levels of discrimination during vector competence studies it remains unclear which of these species were involved in transmission of BTV-8 and to what degree [1]. We examine the potential policy impact of treating these individual species as a single group and then identify key environmental drivers of the timing of each phenological metric for both the group and its constituent species. More specifically, we hypothesise that the four species differ significantly in their phenology and that these responses can be explained by varying responses to environmental variables characterising different habitat and climatic requirements.

In a broader sense, we also address the statistical complications that arise routinely when analysing phenological data. Phenological variables, which refer to the date on which particular events occur, are inherently circular (with Day = 366, or Day = 367 in leap years, being equivalent to Day = 1). Standard statistical methods (such as linear regression, linear mixed models and GLMMs) fail to account for this circularity, and can therefore lead to biased results and potentially to the detection of spurious relationships. We present a hierarchical Bayesian method for dealing with the circular nature of phenological event data by using a natural extension of linear mixed modelling [20]. This method can be implemented using freely available software packages.

## Materials and Methods

### Trapping methods and locations

**Figure 1:**
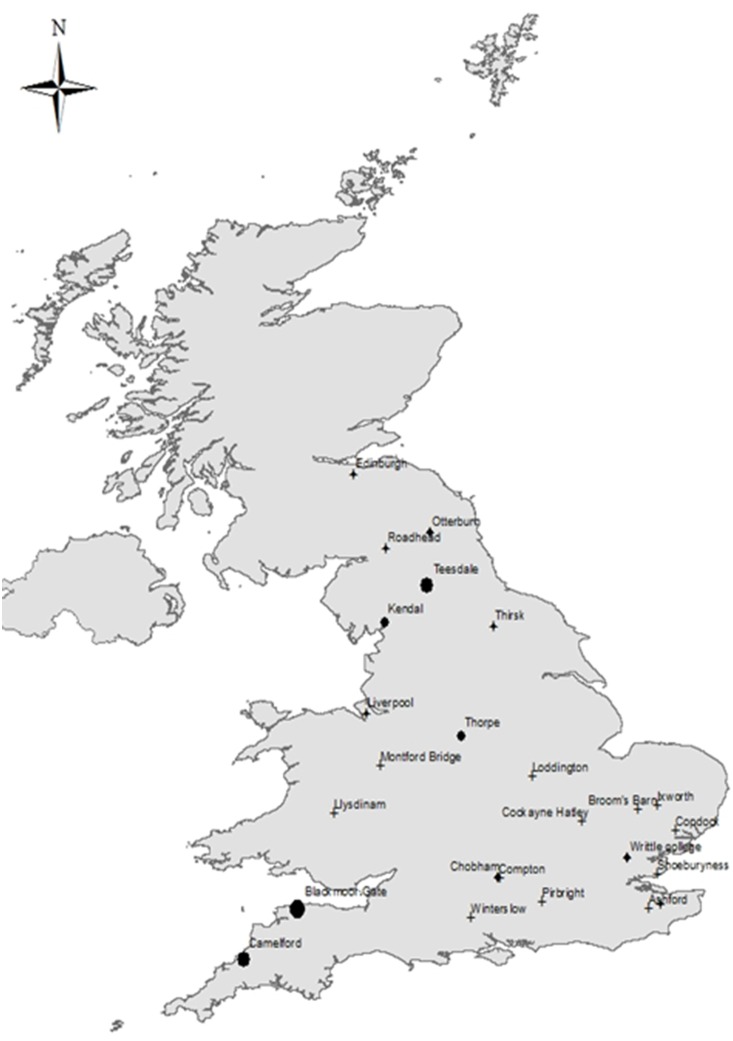
Locations of UK trapping sites for *Culicoides* surveillance (2006–2010). Circle area is proportional to maximum catch ever recorded per site, where complete yearly data were available. Sites with crosses lacked complete yearly trapping profile.

Trapping was conducted from 2006–2010 using standard 8w ultraviolet Onderstepoort Veterinary Institute (OVI) light-suction traps, as previously recommended for *Culicoides* surveillance purposes [21]. OVI traps were hung at approximately 1.5 m as close to livestock as logistically possible to allow permanent positioning and in all cases were ≤25 m distance from ruminant hosts throughout the trapping period. A total of 29 trap locations on private land were used for a variable period of time ranging from one to four years across the UK (Figure 1; for further information regarding the precise location of trapping sites please contact KRS). Access to private land was granted by the landowners. Sampling was carried out by volunteers on one night from dusk until dawn each week with no attempt to synchronise trapping day across the network. Collections were made into approximately 250 ml of water with a drop of detergent to reduce surface tension and then later transferred to 70% ethanol and transported to the Pirbright Institute for identification. Fieldwork did not involve endangered or protected species.

Following receipt, all non-*Culicoides* were removed from the samples based upon morphological features of wing pattern [22] and the absence of humeral pits diagnostic for *Culicoides*. The remaining portion of each sample was categorised into *Culicoides* species groups using wing patterns. Male members of the subgenus *Avaritia* were then identified to species level using genitalia morphology (hereafter referred to as ‘*Avaritia* males’), while females were treated as a single group (hereafter referred to as ‘*Avaritia* females’). Females of the subgenus *Avaritia* were characterised according to the appearance of the abdomen as pigmented, non-pigmented or gravid [23]. Large catches (≥1000 individuals) were subsampled using volumetric methods.

### Timing metrics

From the raw time-series data of weekly trap catches at each site in each year, the date of first appearance (start), last appearance (end), and the length of the inactive overwinter period (length overwinter) were derived for *Avaritia* females and for the individual species in the *Avaritia* male dataset. The start of the season for *Avaritia* females was defined as the first day of the year in which more than five pigmented females were caught (in accordance with the definition used by European Union Council to define the start and end of the SVFP in Europe). Site-by-year combinations were only included in the analysis if at least two trapping nights prior to this date had been recorded. The end of the season for *Avaritia* females was similarly determined as the last day of the year in which more than five pigmented females were caught (with the site-by-year combination being excluded from analysis if there were less than two subsequent trapping nights following this date). For the *Avaritia* males, the start and end of the season were defined as the first (start) and final (end) day of the season when more than five males were caught. The length of the overwinter period was determined as the difference in days between the start of the season in one year, and the end of the season the previous year. Relationships between these three timing metrics and a suite of environmental variables that have previously been found to be important in influencing the seasonal dynamics and abundance of these species were then investigated [24]–[27].

### Environmental covariates

We derived a series of meteorological variables for each site and year combination from the nearest UK Met Office weather station using raw data on total daily precipitation (mm), mean daily temperature (°C), and daily humidity (%) as measured at 15∶00 hrs (Table 1). The mean distance to the nearest weather station was 3.9 km (range: 0.1 km−12.4 km). Photoperiod was calculated daily for each site using latitude and was expressed as the first day of the year at which nine hours of daylight were achieved in each year of observation. Percentage cover of two broad land-cover habitat classes – moorland and heathlands (*moors*) and broadleaf mixed woodland (*brdlf*) - in the surrounding 1 km around each trapping site were determined from the CEH Landcover Map 2007 [28]. The mean densities of cattle and sheep around each trapping site were determined for each year using the Edina AgriCensus data at a 2 km resolution (http://edina.ac.uk/agcensus/).

**Table 1:** Derived meteorological variables from UK Met Office weather station data for each of the trapping site by year combinations used in the timing analyses.

Seasonal metric	Meteorological variable	Definition	Notation
Start of season & lengthof overwinter	Mean wintertemperature (°C)	Mean daily temperatureover November 1^st^ to February 28^th^	T_w_
Start of season	Accumulated degreedays over winter	Accumulated degree daysgreater than 10°Cbetween Novemberand May to capturetemperature variationover the precedingwinter and current spring 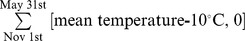	DD_w_
Start of season	Mean springtemperature (°C)	Mean daily temperatureover March 21^st^ to April 30^th^	T_spr_
Start of season	Total springprecipitation (mm)	Summed daily precipitationover March 1^st^ to May 31^st^	P_spr_
Start of season	Mean spring relativehumidity (%)	Mean daily (15∶00 hrs)relative humidity over March 21^st^to April 30^th^.	RH_spr_
End of season	Accumulated degreedays over summer	Accumulated degreedays greater than 10°Cbetween June and Septemberto capture temperaturevariation over the currentsummer and autumn 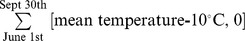	
End of season	Mean summertemperature (°C)	Mean daily temperature over1^st^ to September 30^th^	T_sum_
End of season	Total summerprecipitation (mm)	Summed daily precipitationover June 1^st^ to September 30^th^	P_sum_
End of season	Mean summerrelative humidity (%)	Mean daily (15∶00 hrs)relative humidity overJune 1^st^ to September 30th	RH_sum_

### Statistical models

We used statistical models to analyse relationships between seasonal timing metrics and environmental drivers (see supplementary material for complete details, Section S1). Three different response variables were considered: length of the overwinter period (in days), start of the season (Julian day) and end of the season (Julian day). Analyses were performed for the entire group of *C. obsoletus* complex parous females (*Avaritia* females) and, separately, for each of the individual species within this complex (*Avaritia* males). We constructed sets of environmental variables that were relevant for each seasonal timing metric. The five non-meteorological variables (photoperiod, percent cover of moorland (*moors*), percent cover of broadleaf woodland (*brdlf*), mean density of cattle, mean density of sheep) were assumed to be relevant to all three metrics, but distinct meteorological variables were identified for each seasonal timing metric (Table 1).

Models for length of the overwinter period (days) assumed that the response variable had a normal distribution, whilst models for the start and end of the season were based upon a Wrapped Normal distribution (WN). ‘Start of season’ and ‘end of season’ are circular variables. It would be inappropriate to assume that these variables have a normal distribution because this would imply that the difference between January 1^st^ and December 31^st^ (a difference in Julian days of 364) is twice as large as the difference between January 1^st^ and July 1^st^ (a difference in Julian days of 182). Distributions for circular data deal with this problem by treating the Julian date as a variable that lies on a circle rather than a line, so that the distance between December 31^st^ and January 1^st^ is equal to one rather than 364 [29]. Circular data also arise in other contexts – e.g. when modelling angles and directions. A range of distributions exist for modelling data of this form, but the Wrapped Normal (WN) is commonly used [29]. The WN distribution is symmetric and unimodel, and is obtained by wrapping a normal distribution on the real line around a circle [20]. The WN distribution contains two unknown parameters, which are directly analogous to the mean and variance of the normal distribution. The WN distribution is defined as a distribution for angles, which lie between zero and 2π, and variables that are defined in Julian days therefore need to be multiplied by 2π/365 before the distribution is applied.

We assumed that the mean of the normal distribution (for length of overwinter period) or WN distribution (for start of season and end of season) had a linear relationship with environmental drivers. For *Avaritia* males the linear predictor also included a categorical variable to account for differences between species (as a fixed effect), and allowed for interactions between ‘species’ and the environmental drivers (species-environment interactions). Random effects were also included in models for both males and females in order to account for the structure of the data (and thereby avoid pseudo-replication). When performing analyses for the *Avaritia* females normally distributed random effects were included to account for variation between years (unstructured temporal heterogeneity) and sites (unstructured spatial heterogeneity). The model for *Avaritia* males included a single random effect to capture both site and year effects because the data were highly unbalanced with respect to site and year and so contained little information from which to separate out the effects of site, year and site-by-year interaction (leading to convergence problems in models for the phenological metrics with least data). To check that the use of a single random effect for males was reasonable and did not lead to pseudo-replication, we re-ran the final best-fitting model for the most data-rich phenological metric with additional random effects included (site, year, site-by-species, year-by-species) and found that the inferences regarding environmental relationships and species differences did not change.

### Statistical inference

All models were fitted via Bayesian inference using WinBUGS [30] and the package R2WinBUGS on the R platform [31]. Standard diffuse priors, Normal (0, 100000) for slope parameters and Uniform (0,100) for standard deviations, were assumed for all parameters other than the intercept of the WN model. That parameter is assumed to have a prior of the form Normal (−π,π), because the use of a more diffuse prior may lead to problems with convergence (B. Reich, personal communication). The fitting of the WN model in WinBUGS is not trivial, and we follow the approach of Modlin *et al*. [20]: further details of this approach, along with the BUGS code, are given in Section S2.

Stepwise selection using Deviance Information Criterion [32] was used to select environmental variables (and, for males, species-by-environment interactions) in order to find the most parsimonious model for each seasonal timing metric. DIC is a generalisation of the Akaike Information Criterion (AIC), and is derived as the mean deviance adjusted for the estimated number of parameters in the model – DIC accounts for both model fit and complexity, and providing a relative measure of out-of-sample predictive performance [33]. DIC comparisons to ‘null’ models are presented for all best-fitting models, where the null model contained all fixed and random effects but no environmental variables.

Ideally, the regression models for the start, end and length of the overwinter period should include an estimate of the total abundance of each species at each site in each year. This is because more accurate estimates of the timing of emergence and disappearance are expected at sites with higher abundances of *Culicoides*. However, because of the unequal number and uneven timing of trapping across the sites, we were unable to create a meaningful estimate of overall site abundance for each year within this dataset. This problem is confounded by a degree of circularity between *Culicoides* phenology and abundance. We expect that trapping sites with higher abundance will produce more reliable estimates of the timing of phenological events such as the start and end of the season. However, it is also feasible that the phenology of the species may directly influence abundance, such that sites that are environmentally suitable for earlier spring emergence may also support greater population abundance. Therefore, disentangling phenology from abundance for these species is inherently difficult. To try and reduce this confounding we conducted a second analysis (Section S3) to compare the proportions of the total population of each of the four species that emerged in different seasonal periods for which trapping data were available using the *Avaritia* males dataset (Fig. S3 in Section S3). This analysis allowed us to examine the influence of environmental drivers on the proportion of each species’ population that was active at different periods of the year (Section S3), bypassing the issue of not having a reliable estimate of overall abundance to include in the model. In so doing, we were able to determine that the same environmental variables affected both our measure of phenology, and proportional abundance, thereby providing a measure of the consistency in our inference regarding species-specific phenology (full details in *Section S3: ‘Multinomial analysis of Avaritia males phenology’*).

## Results

Using only those sites and years for which trapping captured the full seasonal profile of *Culicoides* abundance (n = 69, 14 sites over four years), the maximum nightly catch of males per species was 123 (mean 44.1, s.d. 34.8) for *C. chiopterus*, 217 (mean 81.6, s.d. 68.3) for *C. dewulfi*, 460 (mean 86.4, s.d. 116.0) for *C. obsoletus,* and 120 (mean 44.6, s.d. 37.9) for *C. scoticus*. Of these sites, the *Avaritia* male data shows that *C. obsoletus* was caught most frequently (53% of the total maximum catch across all years), followed by *C. dewulfi* (24%), *C. scoticus* (13%) and *C. chiopterus* (10%).

### Start of season

**Figure 2:**
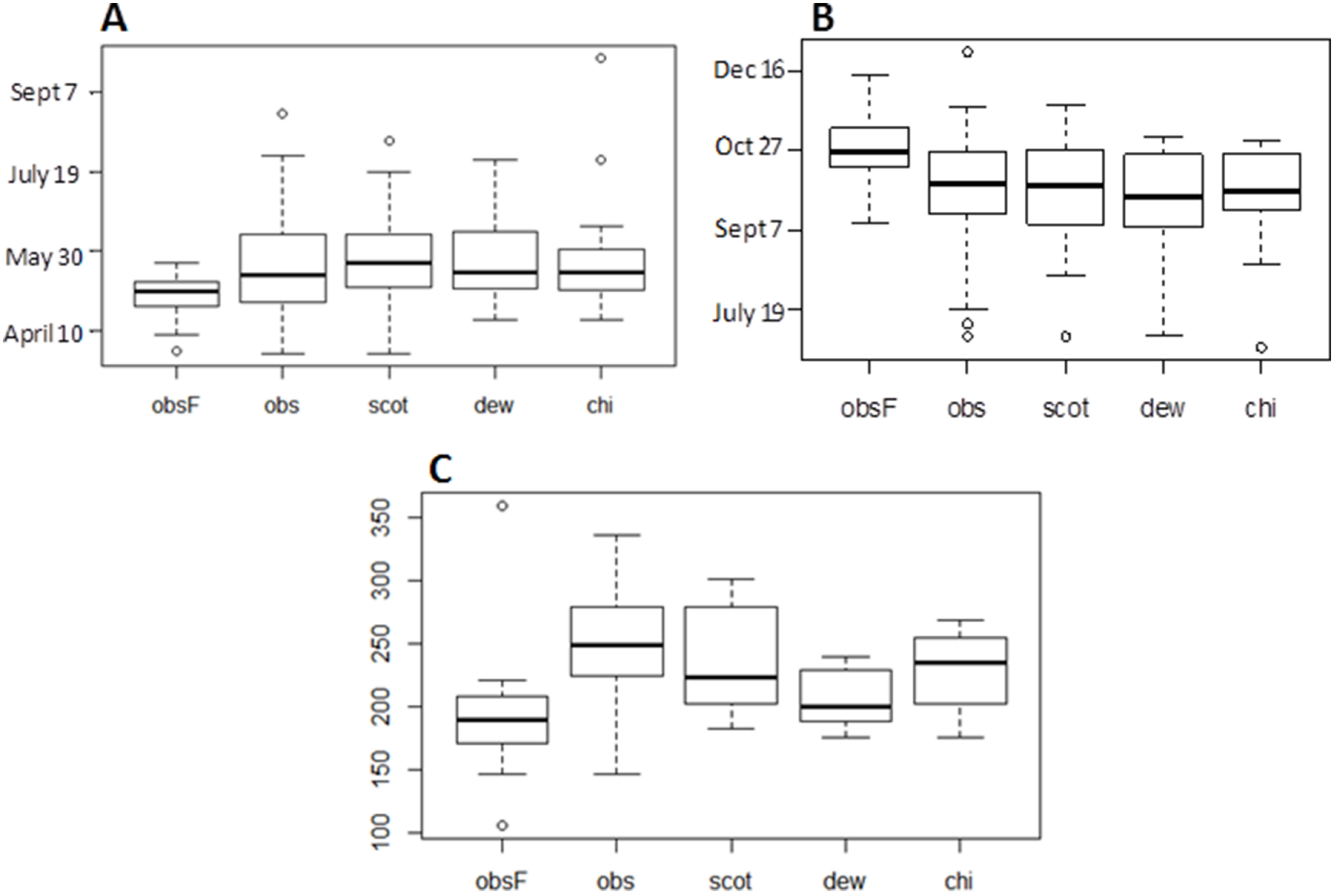
Raw data summarised for the start (A) and end (B) of seasonal activity (date), and length of overwinter period (C; days) derived from UK *Culicoides* surveillance data during 2006 to 2010. Box plots show the median (central line), box denotes 25^th^ and 75^th^ percentiles, error bars represent 10^th^ and 90^th^ percentiles, and dots are points outside the 10^th^ and 90^th^ percentiles. Data are shown for the subgenus *Avaritia* (*Avaritia* females; obsF), *C. obsoletus Avaritia* males (obs), *C. scoticus Avaritia* males (scot), *C. dewulfi Avaritia* males (dew), and *C. chiopterus Avaritia* males (chi).

**Figure 3:**
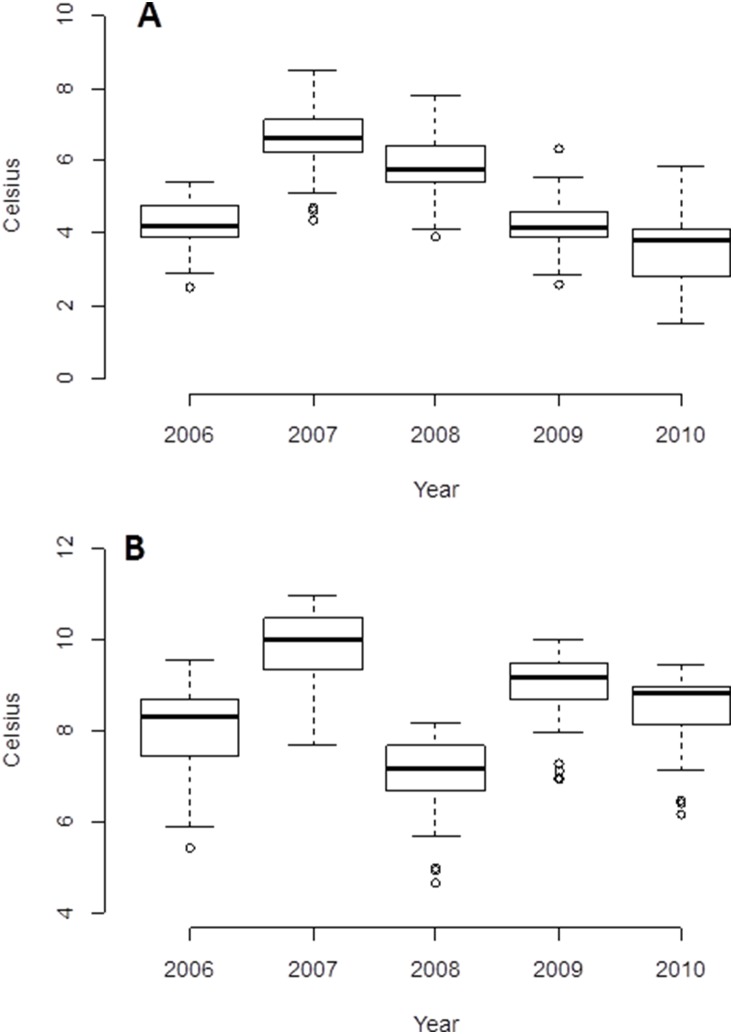
Mean winter (A) and spring (B) temperatures preceding *Culicoides* activity season across all sites for each year of observation. Box plots show the median (central line), box denotes 25^th^ and 75^th^ percentiles, error bars represent 10^th^ and 90^th^ percentiles, and dots are points outside the 10^th^ and 90^th^ percentiles.

Across all sites and years first appearance of *Culicoides* females occurred on average in early May (Table 2, Fig. 2) with the first record in late March (2007, site 3 ‘Chobham’, Fig. 1) and the latest in late May (2009, site 25 ‘Winterslow’, Fig. 1). Average appearance across all sites was remarkably consistent from year to year (early May), with the exception of 2007 in which the *Avaritia* females emerged on average two weeks earlier (Table 2), probably due to the warmer temperatures in winter and spring during that year relative to the other four years of observation (Fig. 3).

**Table 2:** Summary of number of site-by-year combinations observed for each complex or species by timing metric used in the analysis.

								Mean by year				
START	observations	sites	years	Min	Max	Range	Mean	2006	2007	2008	2009	2010
Subgenus *Avaritia*females	49	20	5	88	143	55	123.7	126.3	115.6	127.3	126.1	125.7
*C. obsoletus s.s*	42	18	5	100	236	136	145.4	154.2	140.5	141.8	116.7	165.5
*C. scoticus*	19	10	4 (2009)	114	219	105	150.8	153	150.8	141	-	169
*C. dewulfi*	20	12	5	107	207	100	146.1	163.3	149	133.3	125	129
*C. chiopterus*	17	8	5	107	271	164	150.2	173	158	134.8	139	129
**END**												
Subgenus *Avaritia*females	47	17	5	255	347	92	302	303.6	311.8	297.8	300.6	296
*C. obsoletus s.s*	47	18	5	183	362	179	270.7	275.3	256.6	259	288.8	272
*C. scoticus*	23	10	5	183	328	145	274.6	274.3	255.3	278.3	286.2	276.6
*C. dewulfi*	26	11	5	183	308	125	270.8	267.5	278.5	245.7	294.4	275.2
*C. chiopterus*	24	8	5	229	306	77	278.3	279.5	277.5	262.6	284.3	289.2
**OVERWINTER**												
Subgenus *Avaritia*females	35	16	4	106	221	115	185.4	178.4	184.3	199.8	190.2	-
*C. obsoletus s.s*	26	13	4	147	336	189	247.7	246.3	250.9	235	248	-
*C. scoticus*	8	4	3 (2008)	182	301	119	236.5	219.3	241.3	-	255	-
*C. dewulfi*	8	3	3 (2008)	175	239	64	205.9	201.3	207.3	-	210.5	-
*C. chiopterus*	11	6	4	175	268	93	226.4	234	226.4	235	210.5	-

Years span from 2006–2010, missing years noted in parentheses. Summary of seasonal metrics (minimum, maximum and mean day of the year) across all sites, and mean for each year for *C. obsoletus* group (*Avaritia* females), *C. obsoletus* (*Avaritia* males), *C. scoticus* (*Avaritia* males), *C. dewulfi* (*Avaritia* males), and *C. chiopterus* (*Avaritia* males). START: start of season (day of year), END: end of season (day of year), OVERWINTER: length of overwinter period (days).

Among the subgenus *Avaritia* males, all species emerged in late May, with *C. obsoletus* and *C. dewulfi* tending to emerge on average one week earlier than *C. chiopterus* and *C. scoticus* (Table 2, Fig. 2). The best-fitting model for this metric detected no significant species differences in timing of emergence, however, between *C. obsoletus* and the other three species (Table 3).

**Table 3:** Parameter estimates and 95% credible intervals for the fixed effects for the best-fitting model identified for each seasonal metric for individual subgenus *Avaritia Culicoides* males.

Variable	Estimate (95% credible interval)					
	Overall	*C. scoticus*		*C. dewulfi*	*C. chiopterus*	
Start of season						
Intercept	149.5* (141.0, 158.7)					
Sp		0.22 (−12.82, 13.14)		1.91 (−10.84, 15.43)		−1.82 (−15.91, 12.54)
Cattle	0.32* (0.090, 0.56)					
Cattle:sp		−0.43* (−0.68, −0.18)		−0.43* (−0.68, −0.19)		−0.034 (−0.29, 0.23)
RH_spr_	0.16 (−0.057, 0.38)					
RH_spr_:sp		−0.068 (−0.30, 0.15)		−0.32* (−0.57, −0.059)		0.069 (−0.20, 0.33)
T_spr_	1.94* (0.40, 3.53)					
P_spr_	0.11 (−0.070, 0.29)					
End of season						
Intercept	273.7* (263.6, 283.9)					
Sp		−8.17 (−17.45, 1.56)		−6.31 (−16.14, 3.41)		−3.28 (−13.75, 7.72)
RH_sum_	−2.76* (−5.23, −0.28)					
RH_sum_:sp		1.43 (−0.084, 2.85)		1.62* (0.15, 3.33)		2.38* (0.00081, 4.85)
Sheep	0.024* (0.0058, 0.043)					
Photo	0.31 (−0.067, 0.68)					
Cattle	0.057 (−0.0041, 0.12)					
Length of overwinter period						
Intercept	262.5* (236.3, 286.8)					
Sp			60.09 (−18.53, 144.8)	−30.25 (−140.6, 88.21)		−12.27 (−67.10, 42.30)
Cattle	0.042 (−0.035, 0.12)					
Cattle:sp			−0.15* (−0.25, −0.046)	−0.11 (−0.25, 0.023)		−0.14* (−0.24, −0.029)
Sheep	−0.036* (−0.062, −0.0090)					
Sheep:sp			−0.0032 (−0.052, 0.041)	0.035 (−0.017, 0.085)		0.030 (−0.010, 0.071)
Moors	2.62 (−0.88, 6.23)					

Asterix denotes significance (95% credible interval does not bridge zero). Interactions with species are denoted ‘***:sp’, and the parameter estimates associated with them refer to the differences in effect sizes relative to *C. obsoletus.* The ‘overall’ column contains parameter estimates for main effects (which refer to all species if the corresponding interaction is not present in the model, and refer to *C. obsoletus* if the corresponding interaction is present).

The start of the season for *Avaritia* females was significantly negatively influenced by spring temperature, and close to being significantly positively influenced by photoperiod (more than 94% of posterior density mass greater than zero; Table 4). The best fitting model included both of these variables and received considerably more support in the data than the null model – containing no environmental variables (ΔDIC 4.5, Table S1), although model fit to the data was poor (R^2^ 0.42, including both fixed and random effects).

**Table 4:** Parameter estimates and 95% credible intervals for the fixed effects within the best-fitting model identified for each seasonal metric for the subgenus *Avaritia* female *Culicoides*.

Seasonal metric	Variable	Estimate (95% credible interval)
Start of season	Intercept	123.9* (118.8, 130.9)
	T_spr_	−3.27* (−6.06, −0.44)
	Photoperiod	1.05 (−0.13, 2.22)
End of season	Intercept	296.4* (294.3, 302.3)
	DD_s_	0.031 (−0.0045, 0.064)
Length of	Intercept	184.3* (165.5, 201.9)
overwintering	Photoperiod	2.02 (−1.81, 6.46)
period	Moors	2.83 (−0.54, 6.26)
	Cattle	−0.037 (−0.11, 0.039)

Asterix denotes significance (95% credible interval does not bridge zero).

The best-fitting model for the start of the season for the *Avaritia* males included a significant positive influence of cattle density on the start of seasonal activity for *C. obsoletus* with separate slopes for the other three species showing that start dates for *C. scoticus* and *C. dewulfi* were significantly less delayed by cattle density, and a non-significant difference for *C. chiopterus* (Table 3). A non-significant positive effect of spring humidity for *C. obsoletus* was also identified, with separate slopes for the other three species showing that start dates for *C. dewulfi* were significantly less affected by relative humidity than *C. obsoletus* (Table 3). In addition, a significant positive influence of spring temperature over all species was also determined and a non-significant positive influence of spring precipitation over all species occurred (best-fitting model: R^2^ = 0.73; Table 3). An identical model without spring precipitation received essentially equal support in the data as the best-fitting model (ΔDIC 0.6; Table S2), indicating this variable is perhaps less important than the other three included in the best model. The null model received almost no support in the data in comparison to the best-fitting model (ΔDIC 37.9; Table S2).

### End of the season

Averaged over all sites and years, the last capture date for the *Avaritia* females occurred at the end of October (Table 2, Fig. 2). The earliest final capture occurred in mid-September (2006, site 4 ‘Compton’, Fig. 1), while the latest was in mid-December (2006, site 3 ‘Chobham’, Fig. 1). The four constituent species of the *Avaritia* males had mean dates of last capture within five days of each other, occurring in late September (Table 2, Fig. 2). *C. dewulfi* adults disappeared earliest, followed by *C. obsoletus*, *C. chiopterus* and *C. scoticus* (Table 2, Fig. 2). The best-fitting model for this metric detected no significant species differences between *C. obsoletus* and the other three species (Table 3). Overall, there was greater variability in the end dates for seasonal activity than for the start dates except for *C. chiopterus* (Table 2, Fig. 2).

No significant relationships were detected between environmental variables and the end of the season for *Avaritia* females (Table 4). Moreover, the null model received approximately similar support in the data as all other models, indicating that effects of climate and landcover on this phenological metric were not well captured when modelled as a complex (ΔDIC 1.3, Table S1), and model fit was poor (R^2^ best-fitting model 0.42).

The best-fitting model for the end of seasonal activity for the *Avaritia* males contained a significant negative influence of mean summer humidity for *C. obsoletus* and separate and significantly different slopes for *C. dewulfi* and *C. chiopterus* showing that *C. obsoletus* was inhibited more by summer humidity than both these dung-breeding species (Table 3). A significant positive effect of sheep density was also identified across all species, along with a non-significant positive effect of photoperiod across all species and a non-significant positive effect of cattle density across all species (R^2^ = 0.85; Table 3). Adding mean summer temperature to this model resulted in the same level of support in the data (ΔDIC 0.1, Table S2), as did dropping cattle density (ΔDIC 0.4, Table S2) or dropping photoperiod (ΔDIC 0.6, Table S2). The best-fitting model received considerably more support in the data than the null model (ΔDIC 4.28, Table S2). This suggests the predominant variables influencing the end of the season are summer relative humidity and sheep density; indeed, a model including only these two variables received very similar support in the data as the best fitting model (ΔDIC 0.7, Table S2).

### Length of overwinter

The length of the overwinter period for the *Avaritia* females, when averaged over all sites and years, was approximately 185 days. The shortest overwinter period was 106 days (2006, site 3 ‘Chobham’, Fig. 1), while the longest was 221 days (2006, site 4 ‘Compton’, Fig. 1). The individual species making up the *Avaritia* males showed considerable variation in the length of the inactive overwinter period, with maximum overwinter lengths per species differing by up to 97 days (Table 2, Fig. 2). Averaged over all sites and years, *C. dewulfi* had the shortest overwinter period (206 days), followed by *C. chiopterus* (226 days), *C. scoticus* (237 days), and finally *C. obsoletus* (248 days) (Fig. 2). The best-fitting model for this metric strongly suggested that *C. scoticus* had a longer overwinter period (∼60 days) than *C. obsoletus* (∼93% of the posterior mass for this effect was greater than zero, Table 3), while both *C. chiopterus* and *C. dewulfi* showed no significant difference in overwinter length compared to *C. obsoletus* (Table 3).

The best-fitting model for the length of the inactive overwinter period for the *Avaritia* females included non-significant positive effects of photoperiod and percent cover of moorland and heathland, and a non-significant negative effect of cattle density (Table 4). However, model fit was relatively poor (R^2^: 0.63), and the null model received essentially similar support in the data (ΔDIC 1.1, Table S1).

The best-fitting model for the length of the inactive overwinter period for the *Avaritia* males included a non-significant positive effect of cattle density on *C. obsoletus* with separate slopes for the other three species showing that the length of the overwinter period for all three species was less affected by cattle density than *C. obsoletus,* with both *C. scoticus* and *C. chiopterus* significantly less affected (Table 3). In addition, a significant negative influence of sheep density was demonstrated for *C. obsoletus* with separate, but non-significant slopes for the other three species, and a close to significant positive influence of percent cover of moorland and heathland on all four species (∼93% of posterior density mass greater than zero) (R^2^ = 0.87; Table 3). Several similar models received very close support in the data to the best-fitting model (ΔDIC<2 for next two best-fitting models, Table S2), however, the null model received no support in the data compared to the best-fitting models (ΔDIC 15.8, Table S2).

Variation across years and sites in the phenological models for *Avaritia* females were similar for the start of the season, although there was slightly more variation between years than sites for the end of the season, and slightly less variation between years than sites for the length of the overwinter period (Table S3). For the *Avaritia* males, there was greatest variation across sites and years (combined site*year random effect) for the end of the season, followed by the length of the overwinter and the start of the season (Table S3).

## Discussion

This study has demonstrated that the phenology of *Culicoides* in the UK is species-specific, but also exhibits considerable intra-specific variation between sites and years. The most significant finding in relation to disease control was the documentation of large variations in the length of the inactive overwinter period amongst the constituent species of the subgenus Avaritia. When assessed from catches of males in light-suction traps, *C. dewulfi* had on average the shortest overwinter period (206 days), followed by *C. chiopterus* (∼2 weeks longer), *C. obsoletus* (∼4 weeks longer) and *C. scoticus* (∼8 weeks longer). Importantly, this variation in the overwinter period of males was related to underlying land-cover and host density variables. As such, when defined using data for males, the overwinter SVFP differed by up to eight weeks between the four species. Moreover, evidence from this nationwide surveillance dataset showed that the end of the flight season (autumn) is considerably more variable than the start (spring) across the species comprising the subgenus Avaritia. This is perhaps due to the synchronisation of overwintering larvae into the fourth instar stage resulting in relatively synchronous emergence as adults in the spring. This result implies that accurately predicting the start of the SVFP using current monitoring methods aggregated across species will be difficult. Importantly, when the subgenus *Avaritia* females were modelled as a group, we were unable to detect underlying links with environmental drivers for most of the phenological metrics, indicating that accounting for species-specific variation within this group is important for both understanding phenology and producing models that can be used to predict the SVFP from remote environmental variables.

Models describing the start and end of seasonal activity and the length of the overwinter period for subgenus *Avaritia* males were strongly supported at a species level. Species-specific drivers of phenology identified included increased cattle density (which led to a later start of season in *C. obsoletus*, but had less impact on *C. scoticus* or *C. dewulfi*) and percentage land-cover of moorland (with increasing cover tending to lead to longer overwinter across all species). Additionally, significantly shorter overwintering periods were documented at increased sheep density for *C. obsoletus*, and extended season end dates across all species. *Culicoides obsoletus* also exhibited significantly earlier season end dates at sites with higher mean summer humidity, but that both *C. dewulfi* and *C. chiopterus* were significantly less affected by this variable. *Culicoides dewulfi* was also significantly less influenced by relative humidity in the spring than *C. obsoletus* in relation to the timing of the start of seasonal activity. Warmer spring temperatures also resulted in significantly later start dates for all species using the *Avaritia* male data in direct contrast to the *Avaritia* females, which appeared earlier under warmer conditions.

The biological drivers of these relationships are challenging to interpret, although limited conclusions can be drawn given current knowledge of the ecology of each species. A key consideration is the contrasting types and availability of larval habitat used, with *C. obsoletus* and *C. scoticus* occupying a diverse range of development sites while *C. chiopterus* and *C. dewulfi* are restricted to cattle and horse dung [9], [34]. Somewhat counter-intuitively from an ecological point of view, season start dates for both *C. scoticus* and *C. dewulfi* were significantly less influenced by cattle density than *C. obsoletus*. This is in contrast to our expectations because cattle are known to be an important host for *C. obsoletus*, *C. dewulfi* and *C. chiopterus* [11], [35]. Cattle have been suggested as the most attractive hosts for Palaearctic *Culicoides* [36], [37], though, because hosts have not been enumerated in field studies, a robust host preference has not been conclusively demonstrated at the species level [11]. This observation could arise from interspecific differences in both intrinsic sensitivity of males to UV light and the availability and localisation of mating sites and resting areas. Numbers of *Culicoides* males occurring in UV or incandescent light-suction trap collections usually constitute only a small proportion of the total catch, as seen in both the current study and those conducted across Europe [38]–[40]. No attempt has been made, however, to quantify population responses at a species level in the presence of competing mating cues (e.g. pheromones) or to map resting populations at a farm level scale.

While speculative, similar factors could also drive the observations that greater sheep density resulted in both shorter overwinter periods and significantly later end dates across male populations of all species, although this could also be related to the greater provision of overwinter livestock accommodation of a suitable type to allow later survival of *Culicoides* as documented in northern Europe [10], [11]. Sheep are known to be important hosts for most of these species – *Culicoides scoticus*, *C. dewulfi* and *C. chiopterus* have been collected in abundance on the body of sheep [41], and all four species have been found to engorge on sheep in recent blood meal analysis studies in both farm and extensive pasture settings [35], [37], [42]–[45]. *Obsoletus* group catches in Wales were found to increase with the number of sheep on farms [46], a relationship also identified by a controlled study reporting a linear relationship between *C. obsoletus* trap catches and the number of sheep positioned beneath light traps [47]. It is likely that much finer resolution livestock density data and *C. chiopterus* population data from a passive trapping method would be needed to better understand the relationship and mechanisms whereby host density differentially affects the phenology of these species.

Biological factors underlying climatic drivers of populations similarly require further studies to confirm relationships. In this regard the differential response between dung-breeding species (*C. dewulfi* and *C. chiopterus*) and those utilising less localised and uniform larval development sites is of interest. The localisation of breeding habitats has the potential to lead to restrictions on suitable resting sites for males in order to maximise contact with gravid females. This close association with larval development sites may explain a reduced vulnerability to high humidity levels although adaptation to these conditions in dung breeding species may also play a role. Overall, our results suggest that the resolution and specificity of the freely available climate and ecological datasets we used were not sufficient to accurately decipher how temperature and precipitation interact to drive phenological events in these species, highlighting the need for further work at finer spatial scales.

Importantly, the evidence for considerable variation between species in the length of the inactive period overwinter revealed by analysing data for males in this study has significant implications for disease management via the SFVP in temperate zones. Transmission of vector-borne diseases is highly dependent on the host-vector ratio [48], [49], therefore if these four species are found to be differentially competent for arboviruses, the species-specific variation documented here in terms of their temporal phenology could impact strongly on the length and infection risk of the transmission season for arboviruses in the UK and elsewhere in northern Europe. A key caveat, is that our analysis of male trap catches is an acceptable representation of the phenology of female *Culicoides*. This is important because only female *Culicoides* blood-feed and are responsible for the transmission of viruses. Demonstrating correlation and synchrony in the phenology of males and females of the *Avaritia* species is difficult, and cannot be accomplished without using recently developed high-throughput qPCR assays for pooled samples (which are yet to be proven with collections arising from surveillance). To address this concern with our dataset, we analysed the extent of seasonal correlation in weekly trap catch abundance between males and parous females for two related Palaearctic *Culicoides* species (*C. pulicaris* and *C. impunctatus*) for which we have data spanning the same time period and sites as used in this study (Figs S4a, b in Section S4). This analysis demonstrated that both *C. pulicaris* and *C. impunctatus* exhibited good correlation between the seasonal abundance of male and parous female trap catches with 13 (*C. pulicaris*) and 10 (*C. impunctatus*) of the 15 sites examined for each species showing a correlation of greater than 0.5. In summary, we believe that these data demonstrate good correlation between seasonal trap catches of males and parous females for these two related *Culicoides* species. Moreover, there is no biological reason we are aware of that would suggest a different relationship between male and female seasonal activity for *C. pulicaris* or *C. impunctatus* in comparison to the four *C. obsoletus* complex species used in our main analysis. All of these species require tight synchronisation between male and female seasonal activity to ensure successful reproduction, and this is particularly true in temperate zones such as the UK where multiple generations of these species occur within a single year. Finally, although our analysis cannot define the length of the SFVP for these species because correlation in male and female seasonal abundance and phenology is by no means absolute, it does demonstrate a clear potential for species-specific variation in this important disease management tool. If these findings can be replicated using data on females the implications for disease management and spatio-temporal variation in risk are profound.

A key consideration for the current study and for future surveillance as part of the SVFP lies in the ability in the future to accurately differentiate female members of the subgenus *Avaritia* as these constitute the vast majority of light trap catches. The recent development of high-throughput real-time RT-PCR assays to differentiate species within pools of the subgenus *Avaritia* has great potential in offering a processing method that is sufficiently rapid to sustain surveillance trapping programmes [50]. While the current study has demonstrated that male populations of these species vary in their phenology, uptake of such techniques to examine such variation in females is likely to be determined by the ability to demarcate the role of specific species in the transmission of arboviruses and thereby provide a practical tool for estimating the risk of transmission. While this was not achieved for BTV-8 during the northern European incursion [1], results from the SBV outbreak strongly imply the presence of multiple vector species [51], [52]. This is likely to significantly complicate future attempts to model the risk of transmission according to season. A key observation, however, lay in the observation that the beginning of the adult *Culicoides* flight season was significantly more straightforward to predict than the end. This may enable at least partial prediction of SVFP’s without recourse to costly and time consuming direct surveillance methods.

### Recommendations for management or policy

As part of a surveillance system designed to allow ruminant movements during incursion of *Culicoides*-borne arboviruses, it was suggested that a SVFP could be maintained during which animal movement restrictions could be relaxed. Our study demonstrates that active surveillance of haematophagous female *Culicoides* vector populations cannot currently be replaced using remote models of abundance. This failure was most likely related to the diverse ecology of species conflated within this taxonomic grouping and was partially resolved by the use for species level modelling based on collections of male *Culicoides*.

The differences identified in this study of around eight weeks in the length of the overwinter period for the four species are particularly relevant to disease policy in the UK in relation to defining the SVFP. For instance, the Schmallenberg virus is known to have its greatest impact on mammalian hosts when infection occurs at a particular point in the gestation cycle of the host [5], [18], which coincides with the tail end of the *Culicoides* vector season in the UK. This coincidence in vector phenology and host susceptibility has been demonstrated to drive the extent and size of potential outbreaks of SBV in Scotland [19]. A key finding was that the timing of the end of the season may be more difficult to forecast, and should perhaps be treated with more caution by policy-makers than the beginning of the season, because it varies widely between species, years and locations in response to environmental heterogeneity. We recommend more intensive trapping across a range of climatic zones with species-level identification of *Culicoides* females wherever feasible to facilitate more accurate detection and understanding of the start of the SVFP in temperate zones.

## Supporting Information

Table S1 **Model fit statistics for top ten models identified using forwards and backwards selection with DIC for the start and end of season, and length of overwinter period for the subgenus** ***Avaritia*** **(** ***Avaritia*** **females).** Null model includes only intercept and site and year random effects. The Null model, excluding all environmental effects, is provided for comparison. The difference in DIC between the best-fitting model and each other model is shown by ΔDIC, and *pD* is the effective number of parameters in each model.
(DOC)

Table S2 **Model fit statistics for top ten models identified using forwards and backwards selection with DIC for the start and end of the season, and length of overwinter period for the individual species of the subgenus Avaritia (** ***Avaritia*** **males).** Null model includes intercept, fixed species effect and site*year random effect, and are provided for comparison. The difference in DIC between the best-fitting model and each other model is shown by ΔDIC, and *pD* is the effective number of parameters in each model.
(DOCX)

Table S3 **Parameter estimates and 95% credible intervals for the variances of the random effects within the best-fitting models identified for each seasonal metric for the subgenus** ***Avaritia*** **(** ***Avaritia*** **females and males).**
(DOCX)

Section S1 **Mathematical description of the models used.**
(DOCX)

Section S2 **Bayesian statistical inference for the Wrapped Normal (WN) model.**
(DOCX)

Section S3 **Multinomial analysis of** ***Avaritia*** **males phenology.** File includes: **Figure S3:** Maximum catch size and proportion of the population (based on mean trap catch) trapped in spring (spr), summer (sum) or autumn (aut) months for the four species comprising the subgenus *Avaritia* (*Avaritia* males) across the traps used in the multinomial analysis (chi: *C. chiopterus*, dew: *C. dewulfi*, obs: *C. obsoletus*, scot: *C. scoticus*). **Table S3:** Multinomial results showing posterior means and 95% credible intervals for estimated parameters. Species effects (*sp* and *b_i_sp*) denoted by [2]: *C. scoticus*, [3]: *C. dewulfi*, and [4]: *C. chiopterus*.
(DOCX)

Section S4 **Examination of the correlation in seasonal abundance of male and parous females using data for** ***C. pulicaris*** **and** ***C. impunctatus.*** File includes: **Figure S4a.** Correlation in seasonal trap catches for male and parous female *C. pulicaris* over 15 sites from the UK *Culicoides* surveillance dataset. These 15 site by year combinations had complete seasonal trapping coverage and represent the 15 most abundant site by year combinations in the dataset for this species. **Figure S4b.** Correlation in seasonal trap catches for male and parous female *C. impunctatus* over 15 sites from the UK *Culicoides* surveillance dataset. These 15 site by year combinations had complete seasonal trapping coverage and represent the 15 most abundant site by year combinations in the dataset for this species.
(DOCX)
