# Supplementary material for: Environmental Drivers of Culicoides Phenology: How Important Is Species-Specific Variation When Determining Disease Policy?
Source: PLoS One. 2014 Nov 11;9(11):e111876. doi: 10.1371/journal.pone.0111876 (PMC4227682; doi:10.1371/journal.pone.0111876)
Supplement: Table S1 — Model fit statistics for top ten models identified using forwards and backwards selection with DIC for the start and end of season, and length of overwinter period for the subgenus Avaritia ( Avaritia females). Null model includes only intercept and site and year random effects. The Null model, excluding all environmental effects, is provided for comparison. The difference in DIC between the best-fitting model and each other model is shown by ΔDIC, and pD is the effective number of parameters in each model. (DOC) [file pone.0111876.s001.doc]

**Supplementary Material**

**Table S1.** Model fit statistics for top ten models identified using forwards and backwards selection with DIC for the start and end of season, and length of overwinter period for the subgenus *Avaritia* (*Avaritia* females). Null model includes only intercept and site and year random effects. The Null model, excluding all environmental effects, is provided for comparison. The difference in DIC between the best-fitting model and each other model is shown by ΔDIC, and *pD* is the effective number of parameters in each model.

| **START: Model** | **DIC** | **pD** | **ΔDIC** |
| --- | --- | --- | --- |
| *Null* | *-16.19* | *10.77* | 4.5 |
| **T_spr_+photoperiod** | **-20.65** | **9.88** | **0** |
| T_spr_+RH_spr_ | -20.03 | 10.31 | 0.6 |
| T_spr_+photoperiod+RH_spr_ | -19.71 | 11.04 | 0.9 |
| T_spr_ +RH_spr_+P_spr_ | -19.67 | 11.25 | 1.0 |
| T_spr_ +RH_spr_+P_spr_ + photoperiod | -19.17 | 11.72 | 1.5 |
| T_spr_ | -19.14 | 9.61 | 1.5 |
| T_spr_+P_spr_ | -19.02 | 10.41 | 1.6 |
| Photoperiod+ P_spr_ | -18.92 | 11.25 | 1.7 |
| P_spr_ | -18.50 | 10.16 | 2.1 |
| RH_spr_ | -17.99 | 10.67 | 2.7 |
| **END: Model** | **DIC** | **pD** | **ΔDIC** |
| *Null* | 31.35 | 10.77 | 1.3 |
| **DD_s_** | 30.03 | 10.11 | 0 |
| DD_s_+cattle+*moors* | 30.53 | 11.21 | 0.5 |
| T_sum_ | 30.73 | 10.90 | 0.7 |
| DD_s_+sheep | 30.74 | 10.68 | 0.7 |
| DD_s_+cattle | 30.74 | 10.83 | 0.7 |
| DD_s_+cattle+P_sum_ | 30.81 | 11.49 | 0.8 |
| *moors* | 31.29 | 11.45 | 1.3 |
| DD_s_+P_sum_ | 31.34 | 11.81 | 1.3 |
| DD_s_+photoperiod | 31.42 | 11.98 | 1.4 |
| DD_s_+*moors* | 31.51 | 11.73 | 1.5 |
| **OVERWINTER: Model** | **DIC** | **pD** | **ΔDIC** |
| *Null* | *356.2* | *8.98* | *1.1* |
| **Photoperiod+*moors*+cattle** | **355.1** | **9.52** | **0** |
| Photoperiod+*moors* | 355.7 | 9.22 | 0.6 |
| *moors*+cattle | 356.1 | 9.20 | 1.0 |
| Photoperiod | 356.2 | 9.38 | 1.1 |
| Photoperiod+T_w_ | 356.2 | 9.85 | 1.1 |
| Photoperiod+*moors*+ *brdlf* | 356.2 | 9.67 | 1.1 |
| Photoperiod+ *brdlf* | 356.3 | 9.52 | 1.2 |
| Photperiod+*moors*+T_w_ | 356.4 | 10.04 | 1.3 |
| *moors* | 356.6 | 9.37 | 1.5 |
| T_w_ | 356.7 | 9.61 | 1.6 |
