# Supplementary material for: Environmental Drivers of Culicoides Phenology: How Important Is Species-Specific Variation When Determining Disease Policy?
Source: PLoS One. 2014 Nov 11;9(11):e111876. doi: 10.1371/journal.pone.0111876 (PMC4227682; doi:10.1371/journal.pone.0111876)
Supplement: Table S2 — Model fit statistics for top ten models identified using forwards and backwards selection with DIC for the start and end of the season, and length of overwinter period for the individual species of the subgenus Avaritia ( Avaritia males). Null model includes intercept, fixed species effect and site*year random effect, and are provided for comparison. The difference in DIC between the best-fitting model and each other model is shown by ΔDIC, and pD is the effective number of parameters in each model. (DOCX) [file pone.0111876.s002.docx]

**Supplementary Material**

**Table S2.** Model fit statistics for top ten models identified using forwards and backwards selection with DIC for the start and end of the season, and length of overwinter period for the individual species of the subgenus Avaritia (*Avaritia* males). Null model includes intercept, fixed species effect and site*year random effect, and are provided for comparison. The difference in DIC between the best-fitting model and each other model is shown by ΔDIC, and *pD* is the effective number of parameters in each model.

| **START: Model** | **DIC** | **pD** | **ΔDIC** |
| --- | --- | --- | --- |
| *Null* | *161.54* | *21.88* | *37.92* |
| **Cattle*sp+RH_spr_*sp+T_spr_+P_spr_** | **123.62** | **35.84** | **0** |
| Cattle*sp+RH_spr_*sp+T_spr_ | 124.20 | 35.58 | 0.58 |
| Cattle*sp+RH_spr_*sp+T_spr_+P_spr_+sheep+ *brdlf* | 125.28 | 36.43 | 1.66 |
| Cattle*sp+RH_spr_*sp+T_spr_+P_spr_+sheep | 125.86 | 36.20 | 2.24 |
| Cattle*sp+RH_spr_*sp+T_spr_+sheep | 126.21 | 35.62 | 2.59 |
| Cattle*sp +RH_spr_*sp+*moors**sp +T_spr_+P_spr_ | 126.47 | 40.04 | 2.85 |
| Cattle*sp+RH_spr_*sp | 126.54 | 38.88 | 2.92 |
| Cattle*sp +RH_spr_*sp+*moors**sp +T_spr_ | 127.25 | 39.38 | 3.63 |
| Cattle*sp +RH_spr_*sp+*moors**sp +T_spr_ +P_spr_+sheep | 127.76 | 39.62 | 4.14 |
| Cattle*sp +RH_spr_*sp+*moors**sp +T_spr_+ *brdlf* | 129.23 | 40.71 | 5.61 |
| **END: Model** | **DIC** | **pD** | **ΔDIC** |
| *Null* | *102.5* | *45.27* | 4.28 |
| **RH_sum_*sp+sheep+photoperiod+cattle** | **98.22** | **48.61** | **0** |
| RH_sum_*sp+sheep+photoperiod+cattle+T_sum_ | 98.28 | 48.64 | 0.1 |
| RH_sum_*sp+sheep+photoperiod | 98.63 | 49.16 | 0.4 |
| RH_sum_*sp+sheep+cattle+T_sum_ | 98.78 | 48.47 | 0.6 |
| RH_sum_*sp+sheep | 98.90 | 48.82 | 0.7 |
| RH_sum_*sp+sheep+cattle | 99.06 | 48.51 | 0.8 |
| RH_sum_*sp | 99.83 | 49.06 | 1.6 |
| RH_sum_*sp+sheep+photoperiod+T_sum_ | 99.91 | 48.57 | 1.7 |
| RH_sum_*sp+cattle | 100.00 | 49.04 | 1.8 |
| RH_sum_*sp+photoperiod | 100.18 | 49.43 | 2.0 |
| **OVERWINTER: Model** | **DIC** | **pD** | **ΔDIC** |
| *Null* | *521.47* | *19.91* | 15.8 |
| **Cattle*sp+sheep*sp+*moors*** | **505.67** | **26.53** | **0** |
| cattle*sp+sheep*sp | 506.48 | 25.74 | 0.81 |
| Cattle*sp+sheep*sp+*moors*+*brdlf* | 506.53 | 26.63 | 0.86 |
| cattle*sp+sheep+*moors* | 508.00 | 23.87 | 2.33 |
| cattle*sp+sheep | 508.53 | 23.34 | 2.86 |
| cattle*sp+*moors* | 508.76 | 25.81 | 3.09 |
| Cattle*sp+sheep+*moors**sp | 509.42 | 24.83 | 3.75 |
| Cattle*sp+sheep+*moors**sp+T_w_ | 509.59 | 25.87 | 3.92 |
| Cattle*sp+*moors**sp | 509.98 | 27.09 | 4.31 |
| Cattle*sp+sheep+*moors**sp+ *brdlf* | 510.11 | 25.38 | 4.44 |
