# Supplementary material for: Environmental Drivers of Culicoides Phenology: How Important Is Species-Specific Variation When Determining Disease Policy?
Source: PLoS One. 2014 Nov 11;9(11):e111876. doi: 10.1371/journal.pone.0111876 (PMC4227682; doi:10.1371/journal.pone.0111876)
Supplement: Table S3 — Parameter estimates and 95% credible intervals for the variances of the random effects within the best-fitting models identified for each seasonal metric for the subgenus Avaritia ( Avaritia females and males). (DOCX) [file pone.0111876.s003.docx]

**Supplementary Material**

**Table S3**. Parameter estimates and 95% credible intervals for the variances of the random effects within the best-fitting models identified for each seasonal metric for the subgenus *Avaritia* (*Avaritia* females and males).

| Species | Seasonal metric | Variance estimates (and 95% credible intervals) | | | |
| --- | --- | --- | --- | --- | --- |
|  |  | Site | Year | Year-by-site combination | Residual |
| Females | Start of season | 3.67 (0.20, 8.99) | 3.60 (0.15,14.77) |  | 10.46 (8.24,13.21) |
|  | End of season | 7.22 (3.49,10.27) | 12.20 (4.11,13.77) |  | 17.77 (16.20,19.24) |
|  | Length of overwintering | 17.38 (1.37,37.3) | 8.69 (0.26,35.09) |  | 21.19 (14.69,30.18) |
| Males | Start of season |  |  | 18.97 (11.40,27.05) | 22.24 (18.25,27.25) |
|  | End of season |  |  | 30.12 (22.87,38.48) | 17.39 (14.73,20.72) |
|  | Length of overwinter |  |  | 25.68 (10.41,40.97) | 22.84 (16.15,32.65) |
