# Supplementary material for: Environmental Drivers of Culicoides Phenology: How Important Is Species-Specific Variation When Determining Disease Policy?
Source: PLoS One. 2014 Nov 11;9(11):e111876. doi: 10.1371/journal.pone.0111876 (PMC4227682; doi:10.1371/journal.pone.0111876)
Supplement: Section S1 — Mathematical description of the models used. (DOCX) [file pone.0111876.s004.docx]

**Supplementary material Section S1:**

**Mathematical description of the models used**

We assumed that the mean of the normal distribution (for length of overwinter period) or WN distribution (for start of season and end of season) had a linear relationship with environmental drivers. When performing analyses for the *Avaritia* females this relationship was assumed to be of the form

where $\mu_{st}$is the expected value of the response variable for site *i* in year *j* and $x_{stk}$are the values of the *k*-th environmental variable at site *i* in year *j*. Parameters $\gamma_{t}$ and are random effects that are each assumed to be normally distributed with zero mean and unknown variance

$\begin{matrix} \gamma_{t}\sim\text{N}(0,\sigma_{\gamma}^{2}) \\ \varphi_{t}\sim\text{N}(0,\sigma_{\varphi}^{2}) \end{matrix}$;

these random effects reflect, respectively, unstructured temporal heterogeneity (variation between years) and unstructured spatial heterogeneity (variation between sites). The intercept *a*, regression coefficients *b_k_*, random effect variances σ_γ_^2^  and σ_φ_^2^  and residual variance σ_ε_^2^ are all unknown parameters.

For the analysis of the *Avaritia* males the relationship is assumed to be of the form

$$\mu_{ijk}=a+\alpha_{k}+\sum_{l=1}^{m} (b_{l}+\beta_{kl})x_{ijl}+\delta_{ij}$$

where $\mu_{ij}$is the expected value of the response variable for species *k* at site *i* in year *j*, summed across *l* environmental variables (*x*), and

$$\delta_{ij}\sim\text{N}(0,\sigma_{\delta}^{2})$$

is a random effect reflecting the combined effects of spatial and temporal variation. The additional parameters *α_i_* and *β_ik_* represent species-specific intercepts and species-environment interactions respectively.
