# Supplementary material for: Environmental Drivers of Culicoides Phenology: How Important Is Species-Specific Variation When Determining Disease Policy?
Source: PLoS One. 2014 Nov 11;9(11):e111876. doi: 10.1371/journal.pone.0111876 (PMC4227682; doi:10.1371/journal.pone.0111876)
Supplement: Section S2 — Bayesian statistical inference for the Wrapped Normal (WN) model. (DOCX) [file pone.0111876.s005.docx]

**Supplementary material Section S2:**

**Bayesian statistical inference for the wrapped normal (WN) model**

In order to fit the WN model in WinBUGS it is necessary to introduce an auxiliary variable, *K*, which denotes the “wrap number”. The model for an angle θ is of the form

$\theta\sim\text{WN(}\mu+2\pi K,\sigma_{\varepsilon}^{2})$,

where WN denotes the wrapped normal distribution and μ and σ_ε_^2^ denote the parameters of this distribution. This formulation implies that

$\begin{matrix} \theta|K\sim\text{TN}_{\text{[0,2}\pi\text{]}}\text{(}\mu+2\pi K,\sigma_{\varepsilon}^{2}) \\ P(K=j)=\Phi\left(\frac{2\pi-\{2\pi j+\mu\}}{\sigma}\right)-\Phi\left(\frac{-\{2\pi j+\mu\}}{\sigma}\right) \end{matrix}$ [1]

where TN_R_ denotes a truncated normal distribution over the domain *R* and Φ denotes the cumulative distribution function of a standard normal distribution (see Modlin et al. 2011 for a full derivation). To apply this model within WinBUGS, Modlin et al. (2011) introduced an equivalent representation of [1] such that the prior for K is replaced with a two-stage model that requires only standard parametric distributions, and can be implemented using the ‘ones trick’ (Modlin et al. 2011).

**Model code**

In this section we give WinBUGS code for specifying the Wrapped Normal density for the angle-transformed day of the year for the start and end of seasonal activity for the subgenus *Avaritia* females.

model{

*#priors on fixed regression parameters*

for(i in 1:n){

b[i]~dnorm(0,.0001)

}

*#prior for intercept*

a~dnorm(-3.141592,3.141592)

*#prior for residual variance*

tau <- 1/(sigma*sigma)

sigma~dunif(0,100)

*#prior for random site effect*

for(s in 1:n.site){

epsi[s]~dnorm(0,tau.epsi)

}

*#hyperprior for variance of random site effect*

tau.epsi <- 1/(hsd*hsd)

hsd~dunif(0,50)

*#prior for random year effect*

for(s in 1:y){

yr[s]~dnorm(0,tau.yr)

}

*#hyperprior for variance of random year effect*

tau.yr <- 1/(ysd*ysd)

ysd~dunif(0,50)

for(y in 1:Y){

mu2[y] <- a + epsi[site[y]] + yr[year[y]] + b[1]*cattle[y]

}

for(i in 1:Y){

START[i] ~ dnorm(meany[i],tau)

one[i] <- 1

one[i] ~ dbern(denom[i])

meany[i] <- mu2[i] + 2*pi*K[i]

K[i] <- trunc(-z[i]/(2*pi)) + 1

z[i] ~ dnorm(mu2[i],tau)

U[i] <- phi(sqrt(tau)*(2*pi-mu2[i]))

L[i] <- phi(sqrt(tau)*(0-mu2[i]))

denom[i] <- c/(U[i]-L[i])

}

}End of model

where *c* is a small constant used in the ‘ones trick’ to ensure that denom[i]∈(0,1). The product of the normal density for START (θ_i_) and the Bernouilli density for *one[i]* gives the truncated normal density. For details of the two-stage auxiliary model involving *K* and *z* see Modlin et al. (2011).
