# Supplementary material for: Environmental Drivers of Culicoides Phenology: How Important Is Species-Specific Variation When Determining Disease Policy?
Source: PLoS One. 2014 Nov 11;9(11):e111876. doi: 10.1371/journal.pone.0111876 (PMC4227682; doi:10.1371/journal.pone.0111876)
Supplement: Section S3 — Multinomial analysis of Avaritia males phenology. File includes: Figure S3: Maximum catch size and proportion of the population (based on mean trap catch) trapped in spring (spr), summer (sum) or autumn (aut) months for the four species comprising the subgenus Avaritia (Avaritia males) across the traps used in the multinomial analysis (chi: C. chiopterus, dew: C. dewulfi, obs: C. obsoletus, scot: C. scoticus). Table S3: Multinomial results showing posterior means and 95% credible intervals for estimated parameters. Species effects (sp and bisp) denoted by [2]: C. scoticus, [3]: C. dewulfi, and [4]: C. chiopterus. (DOCX) [file pone.0111876.s006.docx]

**Supplementary Material Section S3:**

**‘Multinomial analysis of *Avaritia* males phenology’**

*Methods*

We calculated the mean number of individuals from each of the four species in the *Avaritia* males dataset that were trapped in the Spring (May-June), Summer (July-September) and Autumn (October-November) at each site for each year (Fig. S3; Winter was not included because of the paucity of trap catches occurring in the winter months). Only sites for which the start and end of seasonal activity were captured and where at least 20 trap catches were recorded between May and November were used in the analysis. We then modelled the mean number of individuals trapped per season for each species as a multinomial regression, where the proportion of the total population trapped per season was derived from linear regressions incorporating environmental effects.

The response vector **Y***_ijks_* for each site *i*, year *j* and species *k* is multinomial, and consists of the mean number of individuals *Y_ijks_* trapped per seasonal period *s*, where *s* = 1 for summer (SUM), *s* = 2 for autumn (AUT) and *s* = 3 for spring (SPR).

$$\begin{matrix} \mathbf{Y}{}_{ijk}\sim\text{ Multinomial}(\mathbf{p}_{ijk},n_{ijk}) \\ p_{ijks}=\frac{\theta_{ijks}}{\sum_{t=1}^{3} \theta_{ijkt}} \\ \log(\theta_{ijks})=a_{s}+\alpha_{k}+\sum_{l=1}^{m} (b_{l}+\beta_{kl})x_{ijl}+\delta_{ij} \end{matrix}$$

where $n_{ijk}=\sum_{s=1}^{3} Y_{ijks}$and log($\theta_{ijk1}$) equals zero for identifiability. The environmental components of the regression equations for $\theta_{ijk3}$and $\theta_{ijk2}$were taken from the best-fitting models for the start and end of seasonal activity for the analysis of the *Avaritia* males dataset described above.

*Results*

We analysed 63 site by year combinations to corroborate the species differences in phenological patterns identified during phase one of the analysis. Environmental relationships and species effects for the proportion of the total population accounted for in spring months were consistent with the previous analysis (start of the season). The effect of cattle density was non-significant, however greater than 75% of the posterior mass was negative (Table S3), suggesting a tendency for sites with greater cattle density to have a lower proportion of the total population occurring in spring months than in the remainder of the year. Greater than 75% of the posterior mass for *C. scoticus* was positive (Table S3), suggesting a lesser influence of cattle density on this species in comparison to *C. obsoletus*, as previously identified in the ‘start of the season’ analysis. None of the remaining three environmental effects (spring humidity, spring temperature and spring precipitation) were significant in this analysis. Greater than 75% of the posterior mass for the parameter controlling individual species response to spring humidity was positive for *C. dewulfi*, suggesting a lesser influence of spring humidity on this species in comparison to *C. obsoletus*, again being consistent with the previous analysis (Table S3). The multinomial analysis for the proportion of the population accounted for during autumn months did not result in any significant relationships.

Although none of the species effects were significant, greater than 75% of the posterior mass for the parameter controlling the proportion of the population occurring in spring months was negative for both *C. scoticus* and *C. dewulfi*, suggesting that a lesser proportion of the population for both of these species occurs in the spring in comparison to *C. obsoletus* (Table S3)*.* This finding suggests that the previous result for *C. scoticus* having a significantly longer overwinter period than *C. obsoletus* may be due to *C. scoticus* tending to have a greater proportion of its population concentrated in the summer and autumn than does *C. obsoletus.*

*Conclusions*

Previous research of *C. punctatus* and *C. pulicaris* species in the UK from 12 suction traps operated daily in England during 2008 has shown that the date of first collection of five or more individuals was influenced by the average population abundance at some sites, with sites of greater abundance having longer periods of seasonal activity [26]. Whether this was due to better detection in traps in more abundant sites is not understood, but this finding is suggestive that the abundance of *Culicoides* may affect our ability to detect the timing of phenological events when they are defined by observations of a small number of individuals. We attempted to account for this potential inaccuracy using a multinomial analysis of the proportion of each population that emerged in different seasonal periods (Fig. S3). The findings from this analysis were consistent with analyses of the individual phenological metric, although much more so for the start of the season than for the end. This suggests that detection probability for cessation in *Culicoides* activity may well be more affected by abundance than for the start of seasonal activity. A true understanding of the relationships between phenology, activity and abundance for *Culicoides* can only be gained from in-depth trapping of identified individuals or cohorts within a population. The inherent difficulties involved in this level of observation mean that we are unlikely to unravel these relationships in natural environments.

**Table S3**. Multinomial results showing posterior means and 95% credible intervals for estimated parameters. Species effects (*sp* and *b_i_sp*) denoted by [2]: *C. scoticus*, [3]: *C. dewulfi*, and [4]: *C. chiopterus*.

| Parameter | SPRING | Variable | AUTUMN | variable |
| --- | --- | --- | --- | --- |
| *a* | -0.039 (-1.30,1.05) | Intercept | -0.92 (-3.14,0.58) | Intercept |
| *b_1_* | -0.97 (-3.28,0.77) | Cattle density | 0.11 (-2.24,2.98) | Summer Humidity |
| *b_2_* | -0.58 (-2.16,0.98) | Spring Humidity | -0.35 (-1.09,0.37) | Sheep density |
| *b_3_* | 0.0079 (-0.76,0.81) | Spring Temp | 0.026 (-0.65,-0.65) | Photoperiod |
| *b_4_* | 0.41 (-0.47,1.32) | Spring ppt | 0.13 (-0.61,0.86) | Cattle density |
| *b1sp* | [2]  0.71 (-1.03,2.93)  [3]  -0.047 (-1.84,2.20)  [4]  0.40 (-1.71,2.95) | Cattle density  *C. scoticus*  *C. dewulfi*  *C. chiopterus* | [2]  -0.52 (-3.86,2.50)  [3]  -0.57 (-3.55,1.86)  [4]  -0.17 (-3.22,2.47) | Summer humidity  *C. scoticus*  *C. dewulfi*  *C. chiopterus* |
| *b2sp* | [2]  0.88 (-0.84,2.75)  [3]  0.76 (-0.78,2.38)  [4]  1.36 (-0.53,3.31) | Spring Humidity  *C. scoticus*  *C. dewulfi*  *C. chiopterus* | NA | NA |
| *spp* | [2]  -0.55 (-2.07,0.88)  [3]  -0.68 (-1.94,0.64)  [4]  -0.12 (-1.57,1.31) | *C. scoticus*  *C. dewulfi*  *C. chiopterus* | [2]  -0.21 (-2.35,2.22)  [3]  -0.077 (-1.90,2.28)  [4]  -0.17 (-2.20,2.28) | *C. scoticus*  *C. dewulfi*  *C. chiopterus* |
| *hsd* | 1.27 (0.34,2.30) | Variance of site*year random effect | 0.31 (0.010,0.94) | Variance of site*year random effect |
| *sigma.o* | 25.00 (1.25,48.77) | Residual variance (of means) | | |

**Figure S3**. Maximum catch size and proportion of the population (based on mean trap catch) trapped in spring (spr), summer (sum) or autumn (aut) months for the four species comprising the subgenus *Avaritia* (*Avaritia* males) across the traps used in the multinomial analysis (chi: *C. chiopterus*, dew: *C. dewulfi*, obs: *C. obsoletus*, scot: *C. scoticus*).
