# Supplementary material for: Environmental Drivers of Culicoides Phenology: How Important Is Species-Specific Variation When Determining Disease Policy?
Source: PLoS One. 2014 Nov 11;9(11):e111876. doi: 10.1371/journal.pone.0111876 (PMC4227682; doi:10.1371/journal.pone.0111876)
Supplement: Section S4 — Examination of the correlation in seasonal abundance of male and parous females using data for C. pulicaris and C. impunctatus. File includes: Figure S4a. Correlation in seasonal trap catches for male and parous female C. pulicaris over 15 sites from the UK Culicoides surveillance dataset. These 15 site by year combinations had complete seasonal trapping coverage and represent the 15 most abundant site by year combinations in the dataset for this species. Figure S4b. Correlation in seasonal trap catches for male and parous female C. impunctatus over 15 sites from the UK Culicoides surveillance dataset. These 15 site by year combinations had complete seasonal trapping coverage and represent the 15 most abundant site by year combinations in the dataset for this species. (DOCX) [file pone.0111876.s007.docx]

**Supplementary Material**

**Section S4. Examination of the correlation in seasonal abundance of male and parous females using data for *C. pulicaris* and *C. impunctatus***

**Methods**

Using data from the same surveillance dataset used in the main analysis, we calculated the correlation between weekly trap catches of males and parous females using data for two related Palaearctic species -- *C. pulicaris* and *C. impunctatus.* We analysed the 15 most abundant site by year combinations for which we had trapping coverage for the entire seasonal period (52 weeks). We restricted our analysis to only the most abundant sites with complete seasonal trapping coverage because of the necessity for a relatively large number of non-zero trap catches for both males and parous females to robustly assess correlation. For each species and site by year combination, we calculated the correlation coefficient (Pearson’s r) over the entire seasonal period between weekly catches of males and parous females.

**Results**

*Culicoides pulicaris* demonstrated good correlation between the seasonal abundance of male and parous female trap catches with 13 out of the 15 sites examined showing a correlation of greater than 0.5 (Fig. S4a). These data encompass eight separate sites and four years.


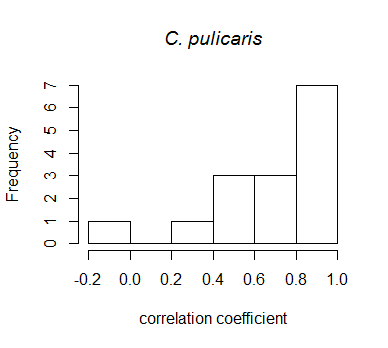


**Figure S4a**. Correlation in seasonal trap catches for male and parous female *C. pulicaris* over 15 sites from the UK *Culicoides* surveillance dataset. These 15 site by year combinations had complete seasonal trapping coverage and represent the 15 most abundant site by year combinations in the dataset for this species.

Similarly, *C. impunctatus* showed good correlation between the seasonal abundance of male and parous female trap catches with 10 of the 15 sites showing a correlation of greater than 0.5 (Fig. S4b). These data encompass six separate sites and six years.


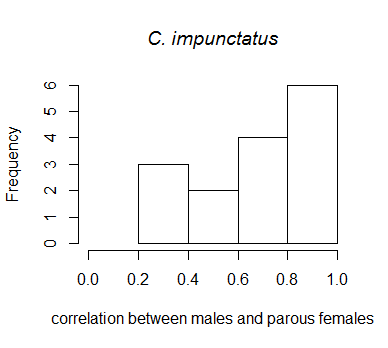


**Figure S4b**. Correlation in seasonal trap catches for male and parous female *C. impunctatus* over 15 sites from the UK *Culicoides* surveillance dataset. These 15 site by year combinations had complete seasonal trapping coverage and represent the 15 most abundant site by year combinations in the dataset for this species.

**Discussion**

In summary, for the 15 most abundant sites in our dataset these data demonstrate good correlation between seasonal trap catches of males and parous females for these two related *Culicoides* species. However, this evidence is not absolute and there is considerable variation in the extent of correlation between males and females across sites and years. This is somewhat attenuated because we have considered correlation across the *entire* seasonal period in this analysis, whereas the main analysis was restricted to only the timing of the start and end of the activity season. As such, we believe this analysis offers sufficient proof to warrant the inference and conclusions made within the main analysis.

It is also important to emphasize that this dataset is very typical of national *Culicoides* surveillance datasets in other EU countries, and as such, is representative of the only viable large scale sources of information on *Culicoides* vector seasonality across Europe. Given the dissuasive practicalities of identifying to species level within datasets on this scale, at present the methodology presented in this analysis remains the only viable alternative to assess if important species differences in phenology exist across vector species.
